# Supplementary material for: PIEZO1 loss-of-function compound heterozygous mutations in the rare congenital human disorder Prune Belly Syndrome
Source: Nat Commun. 2024 Jan 6;15:339. doi: 10.1038/s41467-023-44594-0 (PMC10771463; doi:10.1038/s41467-023-44594-0)
Supplement: Supplementary file 1 — Supplementary Information [file 41467_2023_44594_MOESM1_ESM.pdf]

Amado et al Supplementary information:

Supplementary Figures and Legends:

Supplementary Figure 1

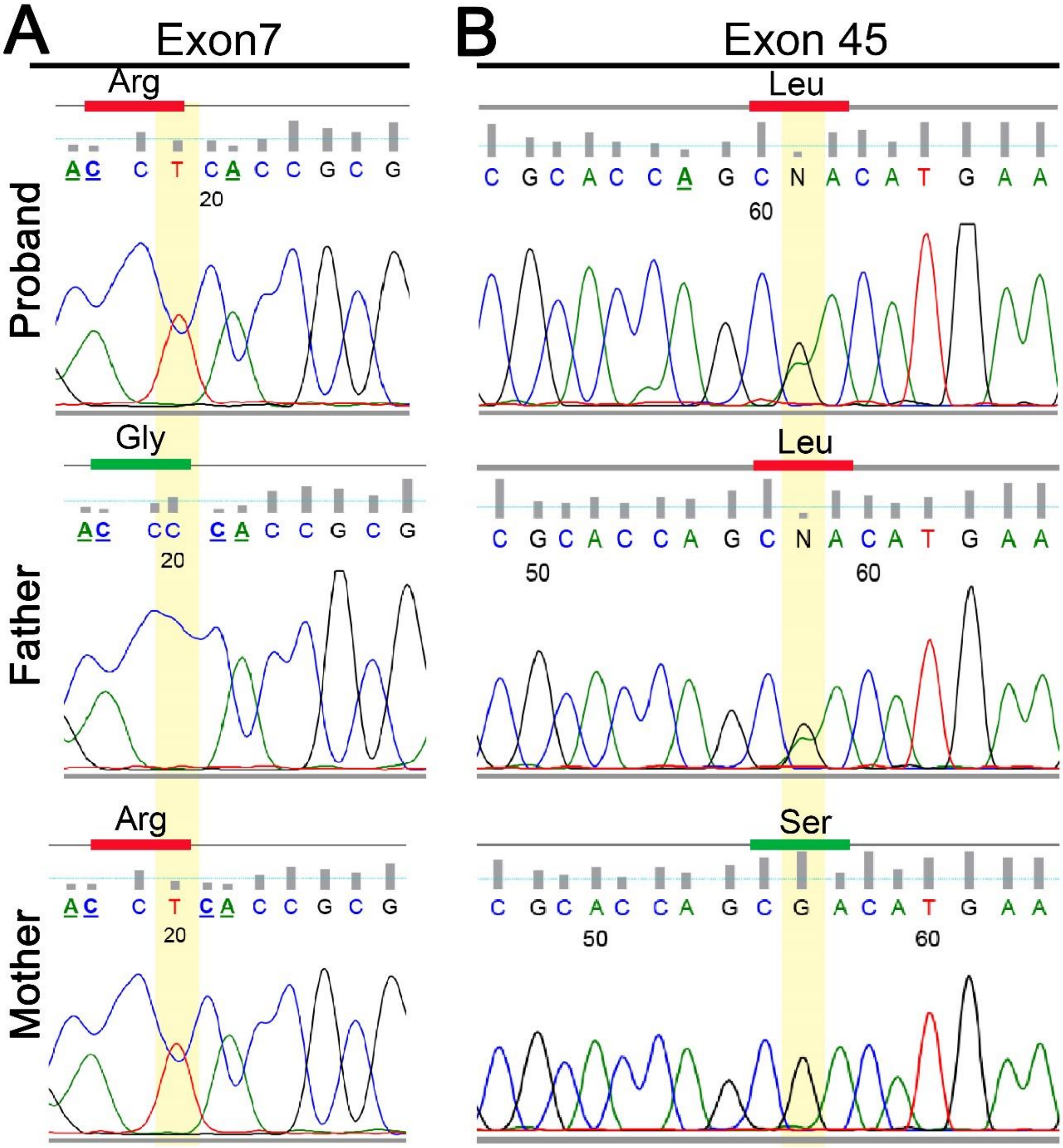

Supplementary Figure 1. Sanger sequencing confirmation of the PBS PIEZO1 variants. (A) Chromatograms of Exon 7 where we identified the c.757G>A (p.Gly253Arg) in the proband and mother. (B) Chromatograms of Exon 45 where we identified the c.6584C>T (p.Ser2195Leu) in the proband and father. The nucleotide variants are indicated in the yellow highlighted regions and the encoded amino acid is indicated in red (wild type) or green (mutant).

Supplementary Figure 2

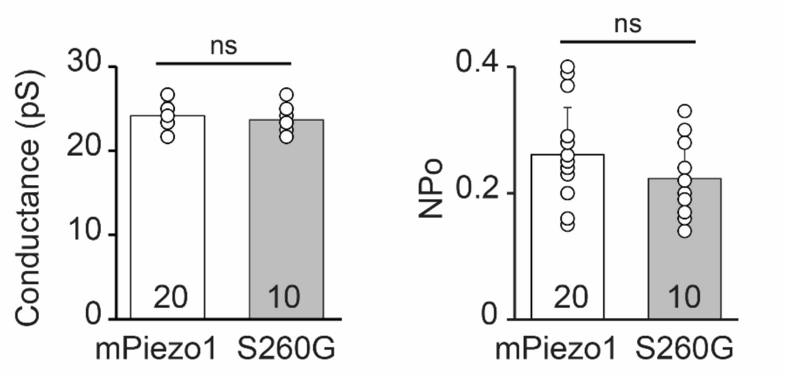

Supplementary Figure 2: Single channel conductance and normalized open probability (NPo) of wild type mouse Piezo1 amino acid S260 (mPiezo1) compared to the wild type human amino acid “G” substitution in mouse Piezo1 (S260G). Number of independent replicates are mentioned in the bar graphs. Statistical analysis Student’s t-test showed no significant difference (ns) $p>0.05$. Data represented as Mean ± SD.

Supplementary Figure 3.

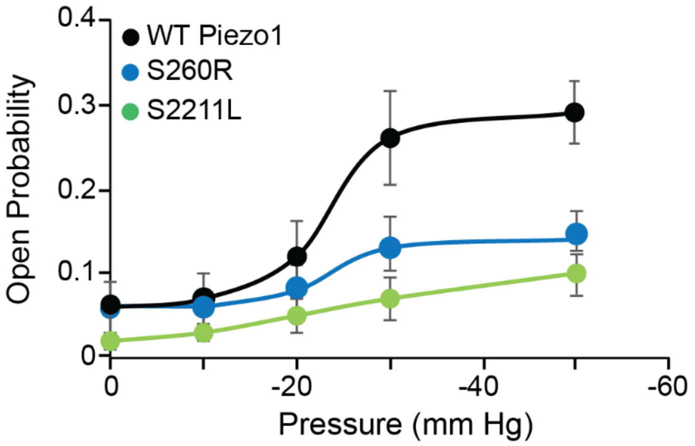

Supplementary Figure 3: Single channel open probability of WT Piezo1 and PBS mutants S260R and S2211L acquired at various pressures (-10 to -50 mm Hg) at a holding potential of -60 mV. Data points represented as Mean ± SD (n >6).

Supplementary Figure 4

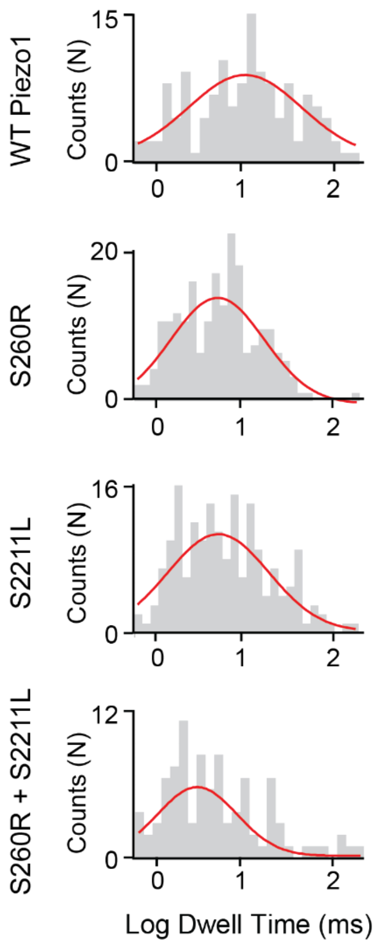

Supplementary Figure 4. Representative Dwell time histograms (n=12), constructed for WT Piezo1, PBS mutants S260R and S2211L, either expressed alone or co-expressed, fitted with the Gaussian curve (red line) to extract mean open time of the channels. The data was acquired at -60 mV and at -30 mm Hg pressure.

Supplementary Figure 5

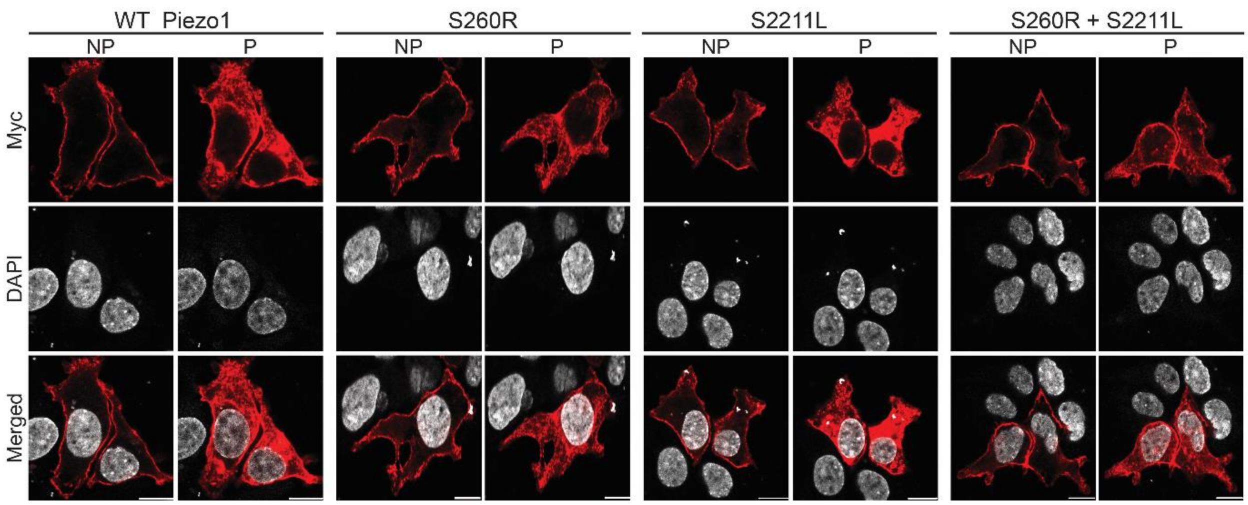

Supplementary Figure 5. Representative images of Myc labeling (n=9) in HEK293T Piezo1 KO cells expressing WT Piezo1, PBS mutants S260R, S2211L and co-expressed S260R+S2211L. Myc tags were introduced in the cap domain of Piezo1 at 2242 amino acid position. Immunostaining was done on fixed cells, with no permeabilization (NP), staining only the surface proteins. Afterwards, the same cells were permeabilized (P) and stained again, staining all of the myc-tagged Piezo1 within the cell. The positive staining of all the construct and qualitative analysis suggests that the mutants of Piezo1 are trafficked to the membrane. Scale bar, 10 µm.

Supplementary Figure 6

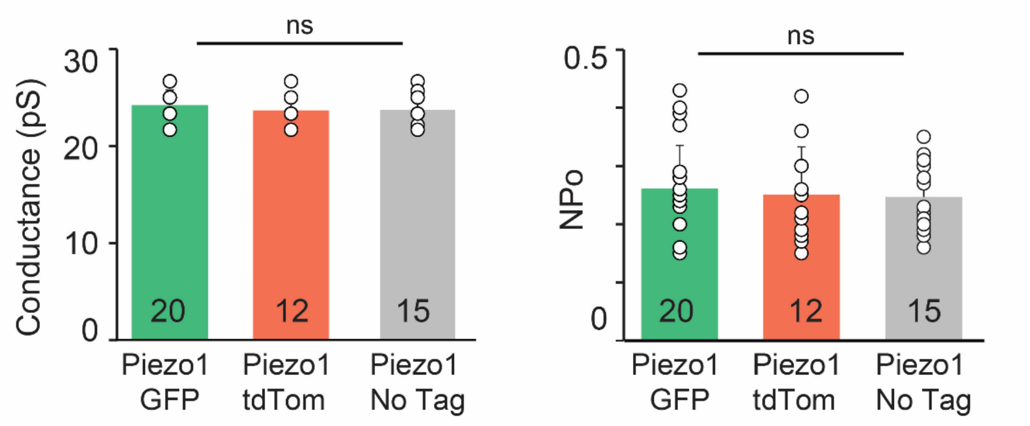

Supplementary Figure 6. Single channel conductance and NPo of WT Piezo1 either fused with GFP, tdTom or without any tag at the C-terminus end. Functional differences were not observed with or without tags as per statistical analysis. T test $p>0.05$ (ns). Data represented as Mean ± SD. Number of independent experiments are mentioned in the bar graphs.

Supplementary Table:

Gene	PolyPhen2	SIFT
GRIN3B; GRAMD1A; TEX36; PCDHGB4; COL6A5; SOX9; CCDC172; NPY4R; SBF2 POLM; UGGT1; COCH; SLC22A12; RNASE1; ADAM11; FAM47C; ADGRL1; ZNF717 LGI4; GPRIN2; NUP214; UBN1; KRTAP4-7; TDGF1	Possibly damaging	Deleterious
CD36; SNAPC4; LEKR1; MCM3; RUNX1; CCDC87; GAREM2; GPR20; SOX10; RYR1; TNS3; IGHMBP2; SDK2; IQGAP2; NEB; SPPL2B; MINDY4; SOX8; COL6A2; TDRD6; PLVAP; SPANXN1; DNAH8; IGLL5; PDZRN3; ADCY9; DOCK3; KRT79; TPSG1; CLNK; GHDC; IL6; LYZL6; NLRP12; TIMELESS; XIRP2; ZNF778	Probably damaging	Deleterious

Supplementary Table 1: Additional variants found by WES on PBS subject. The whole exome sequencing of the PBS subject identified 63 variants in 62 genes, including the compound heterozygous Piezo1 variants. In addition to Piezo1, the variants were filtered by allelic frequency<0.001, SIFT=deleterious, and PolyPhen= damaging or possibly damaging. Note that the only validated variant for the PBS subject was Piezo1, all additional variants on this table were not further confirmed with Sanger sequencing.
